# Antioxidative Strategy for Inflammatory Diseases

**DOI:** 10.1155/2015/675816

**Published:** 2015-05-07

**Authors:** Yung-Hsiang Chen, Ping H. Wang, Pote Sriboonlue, Yuh-Lien Chen

**Affiliations:** ^1^Graduate Institute of Integrated Medicine, College of Chinese Medicine, Research Center for Chinese Medicine & Acupuncture, China Medical University, Taichung 40402, Taiwan; ^2^Department of Medical Research, China Medical University Hospital, Taichung 40447, Taiwan; ^3^Department of Psychology, College of Medical and Health Science, Asia University, Taichung 41354, Taiwan; ^4^University of California Irvine Diabetes Center and Departments of Medicine, Biological Chemistry and Physiology and Biophysics, University of California Irvine, Ivrine, CA 92697, USA; ^5^Department of Biochemistry, Faculty of Medicine, Khon Kaen University, Khon Kaen 40002, Thailand; ^6^Department of Anatomy and Cell Biology, College of Medicine, National Taiwan University, Taipei 10051, Taiwan

Cellular oxidants are unique classes of signaling messengers, those control signal transduction events to drive desirable cellular and biological responses, such as inflammatory cytokine production [1, 2]. However, imbalance oxidative stress causes both cellular damage and biological damage. Thus, it is reasonable to counter balance of oxidative stress to treat inflammation-related diseases by modulating reactive oxygen species (ROS) production. Such antioxidative therapeutic strategies include various endogenous and exogenous antioxidative enzymes, antioxidants, and radical scavengers [3].

This special issue features review articles, original research articles, and clinical studies that portray and expand the current knowledge of the specific antioxidative strategy for inflammatory diseases including therapeutic strategies by modulating oxidative stress in inflammatory diseases, development of antioxidants (enzymes, phytomedicines, nutrients, or radical scavengers) for inflammatory diseases, cellular reduction-oxidation (redox) networks and free radical biology in inflammation, oxidative biomarkers for inflammatory diseases, oxidative stress in immune cell proliferation and death, and redox-sensitive signal transduction pathways (receptor, kinase, and transcription factor) in inflammation.

In this published special issue, we are pleased to present to the reader several articles written by experts in the field. There are three review articles that focus on the role of oxidative stress and inflammation in systemic sclerosis, diabetes, and other various inflammatory diseases, respectively. Given the role of ROS in development of inflammatory diseases, pharmaceutical agents targeting this pathway promise to improve the clinical outcome. These reviews highlight the mechanisms of redox regulation and demonstrate the potential impact of this antioxidative strategy in the management of several acute and chronic inflammatory diseases, including cancer.

In the topic of development of antioxidants for inflammatory diseases, different pharmaceutical agents targeting antioxidative pathway such as rotenone, paeonol, epigallocatechin gallate, *α*-lipoic acid, troglitazones, mitoquinone, serine protease inhibitors, and hydrogen-rich saline were proposed to contribute to detrimental ROS generating processes; it seems to be a reasonable approach to modulate redox pathways in inflammation. In the human organism, a burst in ROS generation is observed during inflammatory diseases. Under pathological conditions with reduced or increased ROS levels different consequences regarding protection or susceptibility to inflammation have to be considered.

In another topic of free radical biology and cellular redox networks (redox-sensitive signal transduction pathways) in inflammation, various redox-related signaling cascades such as interleukin-10, toll-like receptor 4, estrogen receptors, nicotinamide adenine dinucleotide phosphate oxidase, protein kinases, nuclear factor-*κ*B, and nitric oxide synthases were found to play important roles in the regulation of inflammatory diseases. Therapeutic interventions focus on these redox-related signaling cascades which have an impact on the inflammation status and might be utilized as a potential antioxidative strategy in inflammatory diseases. New antioxidative agents designed to scavenge ROS in a redox active state may provide increased efficacy in this regard.

Moreover, for the topic of oxidative biomarkers for inflammatory diseases, oxidative stress makers and vitamin C, cerebrospinal fluid biomarkers, antioxidant profiles (glutathione peroxidase, copper/zinc superoxide dismutase, and glutathione), and lysosomal/membrane enzyme activities indicate an essential involvement of these redox-related biomarkers in acute bacterial osteomyelitis, herpesvirus 6 associated encephalopathy/febrile seizures, and pancreatitis, respectively. These studies suggest that antioxidative system plays the role of the first line of defense against oxidative stress and redox imbalance in the course of acute inflammation.

Taken together, this special issue aims to inspire novel antioxidant/drug development targeting the cellular redox networks to treat inflammatory diseases. The clinicians and researchers summarized the recent ideas with respect to the regulation of oxidative stress and provided the antioxidative therapeutic and delivery strategy for various inflammatory diseases.

## References

[1] Nathan C. (2002). Points of control in inflammation. *Nature*.

[2] Kawaratani H., Tsujimoto T., Douhara A. (2013). The effect of inflammatory cytokines in alcoholic liver disease. *Mediators of Inflammation*.

[3] Sohal R. S., Weindruch R. (1996). Oxidative stress, caloric restriction, and aging. *Science*.

